# Necrolytic migratory erythema and hypoglycemia revealing a pancreatic neuroendocrine tumor

**DOI:** 10.1016/j.jdcr.2026.05.041

**Published:** 2026-05-23

**Authors:** Marine Roux, Aurélie Miot, Damien Boutin

**Affiliations:** a Department of Dermatology, Poitiers University Hospital, Poitiers, France; b Department of Endocrinology, Poitiers University Hospital, Poitiers, France

**Keywords:** glucagonoma syndrome, hyperinsulinemic hypoglycemia, necrolytic migratory erythema, pancreatic neuroendocrine tumor

## Case description

**Fig 1:**
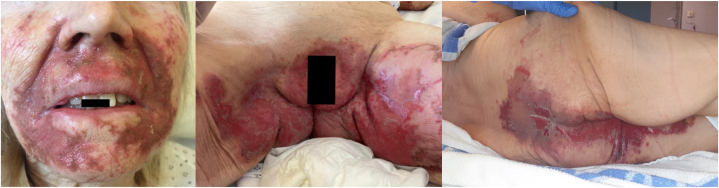
Erythematous, erosive, and scaling plaques involving the perioral area, inguinal folds, and buttocks.

A 75-year-old woman, with no dermatologic history, presented with several months of asthenia, weight loss, depression, and a progressive skin eruption. Lesions initially appeared on the left lower limb before migrating to the gluteal regions, perineum, groins, lower back, and perioral area. Examination revealed erythematous plaques with irregular borders that expanded and coalesced (Fig 1). Initial erythema progressed to desquamation, crusting, and subsequent erosion. These plaques demonstrated centrifugal extension with an active inflammatory border and central healing, leaving residual postinflammatory hyperpigmentation. Lesions were nonpruritic and painless. Glossitis was treated as oral candidiasis; however, no oral or genital mucosal erosions were noted. Edema of the lower limbs led to the diagnosis of bilateral proximal femoral vein thromboses.

**Fig 2:**
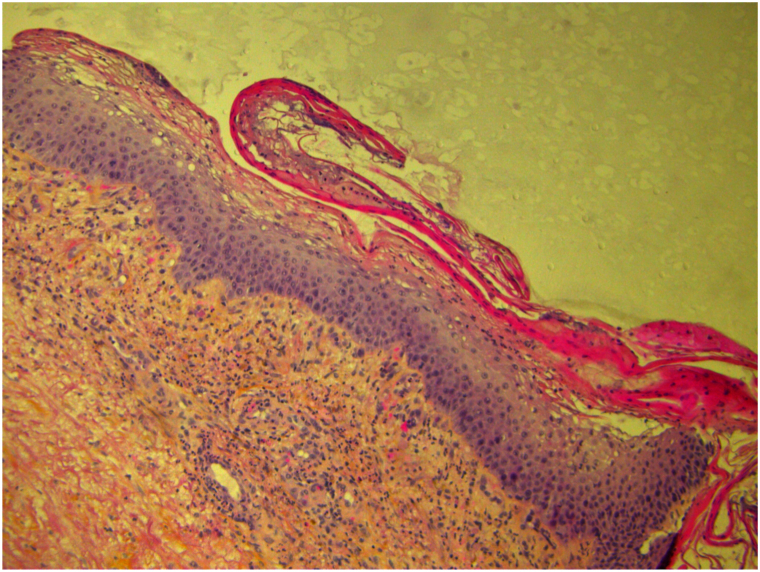
Skin biopsy demonstrating a central pale zone of necrolysis in the upper epidermis, with apoptotic and vacuolated keratinocytes under a superficial layer of parakeratosis (hematoxylin-eosin-saffron stain; ×100).

Skin biopsy demonstrated superficial epidermal necrolysis with vacuolated keratinocytes and a perivascular lymphocytic infiltrate (Fig 2).

Laboratory findings included normocytic anemia (hemoglobin 7.9 g/dL) and multiple micronutrient deficiencies (zinc, folate, selenium, vitamins C and D). Initial nutritional support and zinc supplementation yielded no cutaneous improvement.

Metabolic investigations revealed hypoglycemia, with a lowest venous plasma glucose of 1.76 mmol/L, below the 2.8 mmol/L diagnostic threshold for hypoglycemia in nondiabetic individuals. However, insulin levels were inappropriately normal (102 pmol/L) with elevated C-peptide (1.61 nmol/L; reference: 0.26-1.39 nmol/L) and proinsulin (500 pmol/L; reference: 3.4-20 pmol/L), indicating endogenous hyperinsulinemia. Sulfonylurea screening in serum was negative, excluding drug-induced hypoglycemia. Plasma glucagon was simultaneously elevated (150 pmol/L; reference: 0-60 pmol/L).

**Question: Based on the clinical and histologic findings, what is the most likely diagnosis for this cutaneous eruption?**

**A.** Acrodermatitis enteropathica
**B.** Pellagra
**C.** Necrolytic migratory erythema
**D.** Paraneoplastic pemphigus
**E.** Erythema gyratum repens

## Answer and discussion

Correct answer: **C.** Necrolytic migratory erythema.

**Fig 3:**
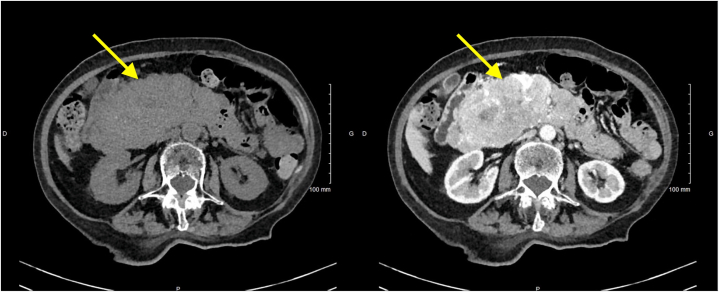
Contrast-enhanced abdominal CT scan showing a large, heterogeneous mass measuring 120 × 150 × 64 mm located in the pancreatic head. Arrows indicates the pancreatic mass. *CT*, Computed tomography.

**Fig 4:**
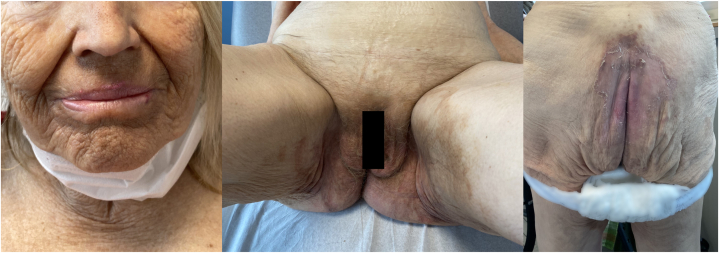
Near-complete resolution of necrolytic migratory erythema after treatment with a somatostatin analog.

Abdominal computed tomography scan in our patient revealed a 12 × 14 cm pancreatic head mass with regional lymphadenopathy (Fig 3). Biopsy confirmed a well-differentiated grade II pancreatic neuroendocrine tumor. Positron emission tomography–computed tomography showed distant nodal metastases. Due to unresectability, medical management with everolimus, diazoxide (for hypoglycemia), and lanreotide (long-acting somatostatin analog) was initiated. The cutaneous eruption significantly improved within 4 weeks of therapy (Fig 4).

Necrolytic migratory erythema (NME) is a cutaneous manifestation of glucagonoma syndrome, a paraneoplastic phenomenon classically characterized by the triad of NME, weight loss, and new-onset diabetes mellitus. Clinically, NME presents as an expanding, annular, erythematous eruption with central blistering and crusting, typically affecting intertriginous and periorificial areas.^1^ Histopathology shows an atrophic epidermis with a central area of pallor resulting from vacuolated and apoptotic keratinocytes below a superficial layer of parakeratosis.^2^

Our case presented NME in the setting of biopsy-proven pancreatic neuroendocrine tumor associated with paradoxical hyperinsulinemic hypoglycemia, rather than classic hyperglycemia. This simultaneous elevation of insulin and glucagon suggests either a tumor co-secreting both hormones or a reactive hyperglucagonemia failing to counteract a tumor-driven insulin excess.^3^^,^^4^ While NME can occur in pseudo-glucagonoma syndromes (eg, severe malnutrition or zinc deficiency),^1^ the lack of response to zinc supplementation and targeted nutritional support and the rapid resolution following tumor-directed therapy (somatostatin analogues)^5^ strongly support a paraneoplastic etiology.

Early recognition of NME is important, as it may be the first sign of an underlying pancreatic neuroendocrine malignancy. Clinicians should consider this diagnosis in patients with characteristic periorificial or intertriginous erosive lesions, even in the absence of hyperglycemia.

## Conflicts of interest

Dr Miot reports support for attending meetings from Ipsen Pharma outside the submitted work. Drs Roux and Boutin have no conflicts of interest to declare.
